# LC-MS- and ^1^H NMR-Based Metabolomics to Highlight the Impact of Extraction Solvents on Chemical Profile and Antioxidant Activity of Daikon Sprouts (*Raphanus sativus* L.)

**DOI:** 10.3390/antiox12081542

**Published:** 2023-08-01

**Authors:** Ciro Cannavacciuolo, Antonietta Cerulli, Verena M. Dirsch, Elke H. Heiss, Milena Masullo, Sonia Piacente

**Affiliations:** 1 Dipartimento di Farmacia, Università degli Studi di Salerno, Via Giovanni Paolo II n. 132, 84084 Fisciano, SA, Italy; ccannavacciuolo93@gmail.com (C.C.); acerulli@unisa.it (A.C.); mmasullo@unisa.it (M.M.); 2 Ph.D. Program in Drug Discovery and Development, Università degli Studi di Salerno, Via Giovanni Paolo II n. 132, 84084 Fisciano, SA, Italy; 3 Department of Pharmaceutical Sciences, University of Vienna, Althanstrasse 14, 1090 Vienna, Austria; verena.dirsch@univie.ac.at (V.M.D.); elke.heiss@univie.ac.at (E.H.H.)

**Keywords:** *Raphanus sativus* L., Brassicaceae, daikon, green extracts, hydroxycinnamic acid derivatives, TEAC, DPPH, Nrf2, multivariate data analysis

## Abstract

Currently, the interest of consumers towards functional foods as source of bioactive compounds is increasing. The sprouts of *Raphanus sativus* var *longipinnatus* (Brassicaceae) are “microgreens” popular, especially in gourmet cuisine, for their appealing aspect and piquant flavour. They represent a functional food due to their high nutritional value and health-promoting effects. Herein, the sprouts of daikon were extracted by different solvent mixtures to highlight how this process can affect the chemical profile and the antioxidant activity. An in-depth investigation based on a preliminary LC-ESI/LTQOrbitrap/MS profiling was carried out, leading to the identification of nineteen compounds, including glucosinolates and hydroxycinnamic acid derivatives. An undescribed compound, 1-*O*-feruloyl-2-*O*-sinapoyl-β-D-glucopyranoside, was isolated, and its structure was elucidated by NMR spectroscopy. The phenolic content and radical scavenging activity (DPPH and TEAC assays), along with the ability to activate Nrf2 (Nrf2-mediated luciferase reporter gene assay) of polar extracts, were evaluated. The results showed the highest antioxidant activity for the 70% EtOH/H_2_O extract with a TEAC value of 1.95 mM and IC_50_ = 93.97 µg/mL in the DPPH assay. Some 50% and 70% EtOH/H_2_O extracts showed a pronounced concentration-dependent induction of Nrf2 activity. The extracts of daikon sprouts were submitted to ^1^H NMR experiments and then analyzed by untargeted and targeted approaches of multivariate data analysis to highlight differences related to extraction solvents.

## 1. Introduction

Functional foods represent a class of food associated with several powerful health benefits. They, besides providing nutrients and energy, are able to modulate targeted functions by enhancing a certain physiological response and/or by reducing the risk of diseases [1]. The sprouts of *Raphanus sativus* var *longipinnatus* (Brassicaceae) are commonly known as ‘kaiware daikon’ in Japanese traditional cuisine [2,3]. This food product belongs to the category of “microgreens”, edible garnishes largely used in gourmet cuisine for visual appeal and piquant flavor [4]. Along with their charm as novel food, microgreens have a high nutritional value and functional effects [5]. In addition to vitamins such as L-ascorbic acid, tocopherols and carotenoids, the occurrence of flavonoids, polyphenols and glucosinolates contribute greatly to the antioxidant activity reported for microgreens [6]. Polyphenolics and glucosinolates from the sprouts of *R. sativus* are reported to reduce oxidative damage and exert cancer-preventive activity [7,8,9]. These reports encouraged a detailed study of the chemical profile, including some biological activities of *Raphanus sativus* var *longipinnatus* (daikon), cultivated in Italy.

Functional foods can be processed to obtain extracts used as dietary supplements with antioxidant properties [10]. The extraction processes are known to affect the chemical profile of the extracts and consequently the occurrence of healthy metabolites as well as the related antioxidant activity [11,12]. Herein, the daikon sprouts were extracted using different solvent mixtures, and a comprehensive phytochemical study based on analytical methods, including Liquid Chromatography–Mass Spectrometry (LC-MS) and Nuclear Magnetic Resonance (NMR), was carried out. To unambiguously identify compounds occurring in the polar extracts of daikon sprouts, a preliminary LC-HRMS (Liquid Chromatography–High Resolution Mass Spectrometry) analysis of MeOH extract which guided the isolation of pure compounds was performed. Isolated compounds were characterized by NMR analysis. The antioxidant activity related to daikon sprouts was previously evaluated for MeOH, CHCl_3_ and CH_3_COCH_3_ extracts [6,13]. Herein, the radical scavenging activity of the MeOH extract and “green” extracts obtained by using EtOH and EtOH:H_2_O (50% and 70% *v*/*v*) mixtures was determined by 1-diphenyl-2-picrylhydrazyl (DPPH) and Trolox Equivalent Antioxidant Capacity (TEAC) assays. To complement data on the direct antioxidant activity evidenced in the DDPH and TEAC assays, all the extracts were further tested by a nuclear factor erythroid 2–related factor 2 (Nrf2)-driven luciferase reporter gene assay. Nrf2 plays a prominent role in the inducible cell defense system, and dietary phytochemicals are reported activate these defense systems [14]. As a stress-responsive transcription factor, it binds to antioxidant responsive element (ARE) consensus sequences in the regulatory regions of target genes, mediating the expression of defensive genes that promote detoxification of chemicals and reactive oxygen species (ROS) and prevent the production of free radicals. Important target genes of Nrf2 include the phase II enzymes heme oxygenase 1 (HMOX1), glutamate-cysteine ligase catalytic subunit (GCLC) involved in glutathione biosynthesis, NAD(P)H dehydrogenase 1 (NQO1), which catalyzes quinone detoxification, and glutathione S-transferase (GSTs) which conjugates xenobiotica or metabolites for facilitated excretion [15,16,17]. With the purpose of comparing the metabolite profiles of the extracts and understanding how the extraction methods could affect the chemical profiles, multivariate statistical analysis (Principal Component Analysis (PCA)) was carried out using data obtained by NMR experiments. Moreover, Partial Least Squares Discriminant analysis (PLS-DA) analysis was carried out to observe the relationship between the chemical profiles of the extracts and their bioactivity profiles.

## 2. Materials and Methods

### 2.1. General Methods

IR spectra were measured on a FTIR IFS-48 spectrometer (Bruker, BioSpinGmBH, Rheinstetten, Germany). NMR experiments were acquired on a Bruker Ascend-600 NMR spectrometer (Bruker BioSpin GmBH, Rheinstetten, Germany) equipped with a Bruker 5 mm PATXI probe. For the isolated compounds, DQF-COSY, HSQC, HMBC and ROESY spectra were acquired in methanol-*d*_4_ (99.95%, Sigma-Aldrich, Milan, Italy), and standard pulse sequences and phase cycling were used. The 1D and 2D NMR data were processed by TOPSPIN 3.2 software. Semipreparative HPLC separations were carried out by using a Phenomenex C18 Synergy-Hydro-RP (250 mm × 10 mm, 10 μm) column on an Agilent 1260 Infinity system (Agilent Technologies, Palo Alto, CA, USA), equipped with a binary pump (G-1312C) and a UV detector (G-1314B). The mobile phase consisted of solvent A (H_2_O + 0.1% formic acid) and solvent B (CH_3_CN + 0.1% formic acid).

LC-ESI/HRMS data were acquired on an LTQ Orbitrap XL mass spectrometer (Thermo Fisher Scientific, San Josè, CA, USA) operating in negative ion mode.

### 2.2. Plant Material and Extraction

*Raphanus sativus* L. var. *longipinnatus* sprouts (1 kg) were purchased by producers in Cesena, Italy, in September 2020. A voucher specimen has been deposited in the Department of Pharmacy of the University of Salerno. The sprouts were frozen at −80 °C for three days and treated by freeze-drying. After 1 night of storage at 4 °C, they were submitted to lyophilization resulting in 150 g freeze-dried sprouts.

A total of 30 g of lyophilized sprouts was submitted to MeOH extraction at room temperature (500 mL of solvent, three-time) to obtain 3.78 g of dried extract, further subjected to *n*-BuOH-H_2_O (50:50) repartition to remove free sugars. A total of 15 mL of H_2_O and *n*-BuOH was used to obtain 1.1 g of *n*-BuOH fraction.

The sprouts of *R. sativus* (3 g) were submitted to extraction with absolute EtOH and EtOH/H_2_O mixtures of 50% and 70% (*v*/*v*) (50 mL for 3 days, three times). The extracts were filtrated and evaporated to dryness in vacuo, and 805.2 mg, 630.3 mg and 1128.2 mg of EtOH and 50% and 70% EtOH/H_2_O of crude extracts were obtained, respectively.

### 2.3. LC-HRMS Profiling

Qualitative LC-MS was performed using a Thermo Scientific Accela HPLC system (Thermo Scientific, Bremen, Germany) equipped with a Luna C18 column (RP-18, 2.0 × 150 mm, 5 µm; Waters; Millford, MA, USA) at a flow rate of 0.2 µL/min and coupled to an LTQ-Orbitrap XL mass spectrometer. Linear gradient elution was carried out by using water with 0.1% formic acid as eluent A and acetonitrile with 0.1% formic acid as eluent B, starting at 10% B. After 25 min, the % B was at 50%, then rising until 100% B at 28 min, remaining at 100% B for 5 min. The autosampler was set to inject 5 μL of each extract dissolved in methanol (1 mg/mL).

The instrument was calibrated using the manufacturer’s calibration standards. The scan was collected in the Orbitrap at a resolution of 30,000 in a *m*/*z* range of 200–1500 amu. The *m*/*z* of each identified compound was calculated to 4 decimal places and measured with a mass accuracy of <3 ppm. The source voltage was −4.0 kV, and the capillary voltage was −35 kV. The tube lens was offset to −126 V, and the capillary temperature was set at 280 °C. The auxiliary gas was set at 20 (arbitrary units), and the sheath gas was set at 10 (arbitrary units). In full LC-ESI/MS experiments, the Total Ion Current (TIC) profile was produced by monitoring the intensity of all the ions produced and acquired in every scan during the chromatographic run. In order to obtain structural information, data-dependent experiments were performed by acquiring MS2 spectra of the most intense ions produced during the acquisition (Table 1).

### 2.4. Isolation of Hydroxycinnamic Acid Derivatives

The *n*-BuOH fraction of *R. sativus* sprouts was chromatographed by semi-preparative RP-HPLC (Reversed Phase-High Performance Liquid Chromatography) coupled to a UV detector set at a wavelength of 330 nm, and a flow rate of 2.0 mL/min was used. A total of 5 mg in a 50 μL sample was injected. For HPLC runs, the gradient used was 0–15 min, from 10 to 33% B; remaining until 20 min at 33% B; 20–21 min, from 33 to 36% B; remaining in isocratic condition until 26 min and reaching 100% B at 28 min.

In this way, compounds **5** (1.2 mg, *t_R_* = 14.5 min), **6** (1.0 mg, *t_R_* = 16.1 min) **7** (1.1 mg, *t_R_* = 16.6 min), **11** (1.2 mg, *t_R_* = 22.9 min), **12** (2.0 mg, *t_R_* = 25.0 min), **13** (2.0 mg, *t_R_* = 20.2 min), **14** (1.1 mg, *t_R_* = 21.2 min), **15** (1.8 mg, *t_R_* = 21.8 min), **16** (1.8 mg, *t_R_* = 25.8 min) and **17** (1.7 mg, *t_R_* = 26.2 min) were isolated.

*3-O-feruloyl-6′-O-sinapoyl-sucrose* (**16**): amorphous, white solid; IR (Infrared Spectroscopy) (KBr): νmax 3430, 1715, 1565, 1250 cm^−1^; ^1^H and ^13^C NMR (methanol-*d*_4_, 600 MHz) data, see Table 1; HR-MS 561.1598 [M-H]^−^, (calcd for C_27_H_29_O_13_, 561.1588).

### 2.5. Determination of Total Phenolic Content

Extracts were analyzed according to the Folin–Ciocalteu (FC) colorimetric method. The extracts were dissolved in MeOH to obtain a concentration of 0.5 mg/mL. Folin–Ciocalteu phenol reagent (0.5 mL) was added to centrifuge tubes containing 0.5 mL of the extract. The content was mixed, and 1 mL of a saturated sodium carbonate solution was added to each tube, followed by adjusting the volume to 10 mL with distilled water. The content in the tubes was thoroughly mixed by vortex and kept at room temperature for 45 min (until the characteristic blue color developed) and then centrifuged at 3000 rpm for 5 min. The absorbance of the clear supernatant was measured at 725 nm on a UV–visible spectrophotometer (Evolution 201, Thermo Fisher Scientific, Milan, Italy). A control without FC reagent and a blank with MeOH were included as a negative control of the assay. The total phenolic content was expressed as gallic acid equivalents (GAE μmol/mg extract, means ± SD of three determinations) calculated by calibration curves (y = 0.0030x + 0.1245, R^2^ = 0.994) [18].

### 2.6. Determination of DPPH Radical Scavenging Activity and TEAC Radical Scavenging Activity

The radical-scavenging activity of extracts and vitamin C (positive control) was determined by quenching the stable 1,1-diphenyl-2-picrylhydrazyl radical (DPPH^•^). An aliquot (37.5 μL) of the MeOH solution containing different amounts of the extract or vitamin C was added to 1.5 mL of freshly prepared DPPH^•^ solution (0.025 g/L in MeOH). An equal volume (37.5 μL) of the vehicle solvent was added to the negative control tubes. Determinations were repeated three times for each extract. Absorbance at 517 nm was measured on a UV–visible spectrophotometer (Evolution 201, Thermo Fisher Scientific, Milan, Italy) 10 min after the reaction started. The DPPH^•^ concentration in the reaction medium was calculated from a calibration curve (range = 50–200 μg/mL) analyzed by linear regression (y = 0.3203x + 18.9908, R^2^ = 0.991) [19].

TEAC assay was performed on the extracts [20]. The radical scavenging activity of the extracts was determined by the capacity to scavenge the radical cation 2,2′-azinobis(3-ethylbenzothiazoline-6-sulfonate) ABTS^•+^ by spectrophotometric analysis. The extracts were diluted with MeOH to produce solutions of 250, 500, 750 and 1000 µg/mL; the 6-hydroxy-2,5,7,8-tetramethylchroman-2-carboxylic acid (Trolox) was diluted with MeOH to produce solutions of 0.3, 0.5, 1 and 1.5 mM. The reaction was initiated by the addition of 1.5 mL of diluted ABTS to 15 μL of each sample solution. Determinations were repeated three times for each extract. The inhibition percentage of absorbance at 734 nm was calculated for each concentration relative to a blank absorbance (MeOH) and was plotted as a function of the concentration of standard Trolox. The TEAC value was defined as the concentration (mM) of a standard Trolox solution with the same antioxidant capacity as 1 mg/mL of the tested extract [21,22].

### 2.7. Activation of the Nrf2 Pathway Assessed by a Reporter Gene Assay

The luciferase assay was performed as reported by Cannavacciuolo et al. (2022) [23]. Stably transfected cells with HepG2-ARE-Luc (Signosis, Santa Clara, CA, USA, SL-0046-FP) were cultivated at 37 °C and 5% CO_2_ atmosphere in Dulbecco’s modified Eagle’s medium (DMEM; Lonza, Basel, Switzerland), supplemented with benzylpenicillin (100 U/mL), streptomycin (100 μg/mL), glutamine (2 mM) and 10% fetal bovine serum (FBS). Before performing the assay, cells were stained for 1 h in serum-free medium supplemented with 2 μM Cell Tracker Green CMFDA (Invitrogen, Waltham, MA, USA), a fluorescent probe used as an indicator for viable cell number. Cells were then plated in 96-well plates (2 × 10^4^ cells/well) and incubated overnight. The following day, cells were treated with test samples at concentrations of 30, 20, 10 and 5 µg/mL dissolved in DMSO 0.1%. DMSO (final concentration of 0.1%) was used as a negative control, and iberin (3 µM) was used as a positive control. After incubation for 16 h, cells were lysed with a luciferase lysis buffer (Promega, Madison, WI, USA; E1531), and the luminescence of the firefly luciferase and the fluorescence of the Cell Tracker Green CMFDA were measured by a Tecan Spark**^®^** microplate reader (Männedorf, Switzerland). For quantification of induced Nrf2 activity by the test samples, the luciferase-derived signal from the Nrf2-dependent reporter gene was normalized by the fluorescence signal derived from Cell Tracker Green CMFDA to account for potential differences in cell number and then referred to the DMSO only control. Potential cytotoxicity of the test samples was checked by direct comparison of the Cell Tracker Green CMFDA fluorescence of the solvent vehicle-treated cells and cells treated with the investigated samples.

### 2.8. Statistical Analysis

Each LC-HRMS experiment was performed in triplicate. The variance (ANOVA) and *t*-test were applied to estimate differences (considered to be significant at *p* ≤ 0.05). Microsoft Excel 2016 was used for statistical analyses.

### 2.9. ^1^H NMR Parameters and Data Processing for Multivariate Data Analysis

All NMR spectra of the extracts were acquired in methanol-*d*_4_ (99.95%, Sigma-Aldrich) at 24 °C. For each ^1^H NMR experiment, the following parameters were used: number of total scans 80, 3.0 s for relaxation delay, 3.3 s for acquisition time, 64 k data points. An exponential window function with a line broadening factor of 0.2 Hz was applied. Each sample was acquired in triplicate, in order to obtain three ^1^H NMR spectra for each sample.

To analyze the spectra, MestreNova 10 software was used; in particular, each NMR spectrum was manually phased, and baseline correction was applied. To calibrate spectra, a signal of solvent at *δ* 3.34 was selected; in this way, a good peak alignment was obtained. Bucketing in the region 0.5–8.5 ppm region (spectral buckets of 0.04 ppm) was chosen, and the signals of methanol-*d*_4_ and water were excluded. All NMR data were normalized considering total sum normalization. For the targeted metabolite, a key signal was selected. The spectra, converted to ASCII format, were submitted to multivariate data analysis.

For HSQC was selected a spectral width of 12 ppm (proton) and 160 ppm (carbon) with 1 K data points, 32 scans, 256 t1 increments and a recycle delay of 2 s. HMBC was acquired with a spectral width of 12 ppm (proton) and 230 ppm (carbon), 4 K data points, 56 scans, 256 t1 increments and a recycle delay of 2 s.

### 2.10. Multivariate Data Analysis (MVDA)

For metabolomics multivariate data analysis, PCA and PLS-DA were applied [21]. For MDVA, the rows were the area of different metabolites in the ^1^H-NMR dataset (variables), while the columns were represented by different extracts of *R. sativus* (observations).

#### 2.10.1. Principal Component Analysis

The chemical shifts obtained from ^1^H NMR experiment were considered. Untargeted PCA was acquired to achieve general information and to highlight possible trends and outliers among the extracts (observation). For the targeted analysis, a matrix was obtained considering specific chemical shifts ascribable to metabolites (variables); in this way, 20 variables and 4 observations corresponding to the extracts were considered.

For processing, SIMCA P + software 12.0 (Umetrics AB, Umea, Sweden) was used. The matrix, after log transform and Pareto scaling, was analyzed by PCA to define a homogeneous cluster of samples.

#### 2.10.2. Partial Least Squares-Discriminant Analysis (PLS-DA)

PLS-DA was used with the aim of obtaining a discriminant classification; in particular, in PLS-DA [24], the classes were attributed based on different Nrf2 activations, specifically −1 to samples 50% and 70% EtOH/H_2_O (showing higher activity), 0 to EtOH extract (showing mild activity), +1 to MeOH extract (showing lower activity).

Projection methods in Multivariate Data Analysis were performed using SIMCA-P+ software (Version 12.0, Umetrics, Umea, Sweden). To minimize false discoveries and to obtain robust statistical models cross-validation, techniques and permutation tests were used following standardized good practice.

## 3. Results and Discussion

### 3.1. LC-ESI/LTQOrbitrap/MS Profiling of R. sativus Sprouts

To achieve a preliminary overview of MeOH extract of *R. sativus* sprouts, LC-HRMS analysis was performed. The accurate study of the LC-HRMS profile suggested the presence of multiple classes of metabolites. In particular, the analysis of the accurate masses, characteristic fragmentation patterns, retention times and comparison with the literature data on *R. sativus* sprouts [8,25,26] allowed us to tentatively identify 19 main peaks corresponding to glucosinolates (**1**–**4** and **8**–**10**), hydroxycinnamic acid derivatives (**5**–**7** and **11**–**17**) and oxylipins (**18** and **19**). In detail, the negative LC-ESI/LTQOrbitrap/MS of peaks **1**–**4** and **8**–**10** displayed in MS/MS spectra product ions (e.g., product ions at *m*/*z* 275, 259, 241 and 195) originating from rearrangements and breakdown involving the common structural skeleton and formed by neutral loss of moieties of these structural common parts providing the identification of the variable side chains [27,28]. Consequently, compounds **1***–***4** and **8**–**10** were identified as the methionine-like glucosinolates glucoraphenin (**1**), glucoerucin (**3**) and glucoraphasatin (**4**), already reported in the species [25], the indolic glucosinolates hydroxyglucobrassicin (**2**) and methoxyglucobrassicin (**8**) along with the phenolic glucosinolate sinapoylglucoraphanin (**9**) herein reported for the first time in the species. By the LC-MS profile, peak **10** was putatively attributed to benzoylglucoraphanin; in detail, compound **10** displayed a [M-H]^−^ ion at *m*/*z* 538.0502 corresponding to C_19_H_24_O_11_NS_3_. The MS/MS spectrum of compound **10** was characterized by the presence of the product ion at *m*/*z* 523 corresponding to the loss of a methyl group and at *m*/*z* 395, *m*/*z* 379, *m*/*z* 363 corresponding to the different transfer of the sulfate group on glucose, representing typical glucosinolate fragmentations [27].

**Figure 1:**
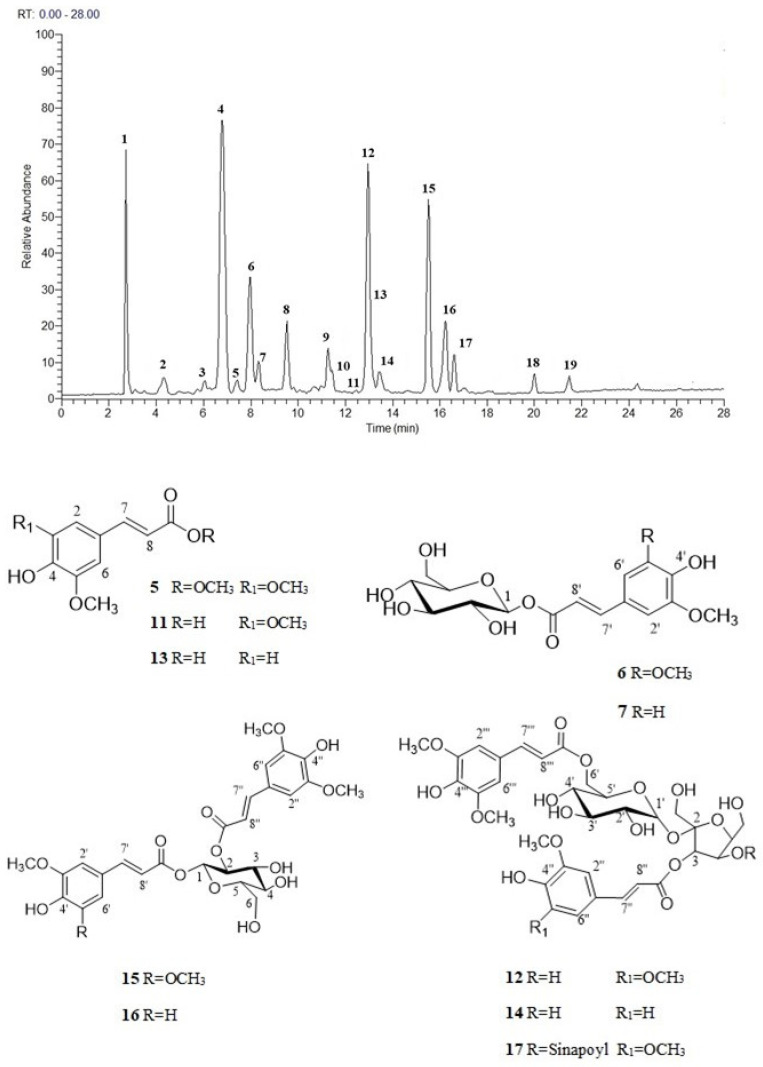
LC-HRMSMS profile in negative ionization mode of MeOH extract of daikon sprouts and structures of some isolated compounds.

Peaks **5**–**7** and **11**–**17** showed in the MS/MS analysis a fragmentation pattern ascribable to hydroxycinnamic acid derivatives. Among these, compounds **6**, **7**, **12** and **14**–**17** showed product ions derived by neutral loss of 162 Da corresponding to a hexose unit suggesting the presence of glycosylated derivatives [29]. Finally, peaks **18** and **19** showed typical fragmentation patterns ascribable to the oxylipin class [30] (Figure 1 and Table 1). To the author’s knowledge, glucosinolates and hydroxycinnamic acid derivatives represent characteristic markers for *R. sativus*; notably, oxylipins are described here for the first time in this species.

**Table 1:** Metabolites putatively identified in the MeOH extract of *R. sativus* sprouts.

	*t_r_*	*m/z*	*ppm*	Molecular Formula	MS/MS	Compound
**1**	2.72	434.1692	−0.64	C_12_H_21_O_10_NS_3_	419, 354, 275, 259, 241, 195	glucoraphenin ^1^
**2**	4.31	463.0468	−1.95	C_16_H_20_O_10_N_2_S_2_	301, 285, 267, 198	hydroxyglucobrassicin ^1^
**3**	6.08	420.1303	−1.26	C_12_H_23_O_9_NS_3_	275, 259, 195	glucoerucin ^1^
**4**	6.84	418.0292	−0.40	C_12_H_21_O_9_NS_3_	338, 275, 259, 241, 175	glucoraphasatin ^1^
**5**	7.28	237.0758	0.41	C_12_H_14_O_5_	237	methylsinapate ^2^
**6**	7.98	385.1130	0.23	C_17_H_22_O_10_	265, 247, 223, 205	1-*O*-sinapoyl-β-D-glucopyranoside ^2^
**7**	8.11	355.1024	0.11	C_16_H_20_O_9_	217, 193, 175	1-*O*-feruloyl-β-D-glucopyranoside ^2^
**8**	9.53	477.0630	−1.91	C_17_H_22_O_10_N_2_S_2_	397, 299, 281, 315, 275, 259, 235, 195	methoxyglucobrassicin ^1^
**9**	11.41	640.1726	−1.08	C_23_H_31_O_14_NS_3_	625, 465, 434, 325, 283, 223	sinapoylglucoraphanin ^1^
**10**	11.53	538.0502	−0.68	C_19_H_25_O_11_NS_3_	523, 379, 376, 363, 282, 256, 241, 224, 201, 192, 169, 156, 136, 129, 121, 97	benzoylglucoraphenin ^1^
**11**	12.94	223.0607	2.81	C_11_H_12_O_5_	164	sinapic acid ^2^
**12**	12.94	753.2516	−2.01	C_34_H_42_O_19_	547, 529, 365, 325, 223	3,6’-*O*-disinapoylsucrose ^2^
**13**	13.21	193.0502	2.51	C1_0_H_10_O_4_	149, 134	ferulic acid ^2^
**14**	13.41	723.2121	−1.37	C_33_H_40_O_18_	547, 529, 517, 499, 337	3-*O*-feruloyl-6′-*O*-sinapoyl-sucrose ^2^
**15**	15.53	591.1705	−0.62	C_28_H_32_O_14_	385, 367, 352, 247, 223, 205	1,2-*O*-disinapoyl-β-D-glucopyranoside ^2^
**16**	16.26	561.1598	−1.01	C_27_H_30_O_13_	367, 337, 284, 223	1-*O*-feruloyl-2-*O*-sinapoyl-β-D-glucopyranoside ^2^
**17**	16.60	959.2794	−0.91	C_45_H_52_O_23_	753, 735, 529, 368	3,4,6′-*O*-Trisinapoylsucrose ^2^
**18**	20.01	327.2167	0.43	C_18_H_32_O_5_	309, 291, 229, 211, 171	9,12,13-trihydroxyoctadeca-10-15-dienoic acid ^1^
**19**	21.48	329.2322	0.06	C_18_H_34_O_5_	311, 299, 211, 171, 139, 127	9,12,13-trihydroxyoctadeca-10-enoic acid ^1^

^1^ compounds putatively identified by LC-ESI/HRMS analysis, ^2^ compounds unambiguously identified by isolation and NMR analysis.

### 3.2. Isolation and Characterization of Hydroxycinnamic Acid Derivatives in R. sativus Sprouts

In order to unambiguously determine hydroxycinnamic acid derivatives in *R. sativus* sprouts, the *n*-BuOH fraction was submitted to HPLC-UV analysis affording compounds **5**–**7** and **11**–**17** (Figure 1). Compounds were analyzed by ^1^H and 2D NMR experiments, and their identity was confirmed by ESI/HRMS analysis.

The ESI/HRMS spectrum of **16** (*m*/*z* 561.1598 [M-H]^−^, calculated for C_27_H_29_O_13_, 561.1588) supported a molecular formula of C_27_H_30_O_13_. The MS/MS spectrum of this ion showed fragment ions at *m*/*z* 367.2461 [M-H-194]^−^, due to the loss of a feruloyl unit, and at *m*/*z* 337.1253 [M-H-224]^−^ attributable to the loss of a sinapoyl moiety. The ^13^C NMR spectrum showed carbon signals attributable to the aglycone moiety and to a sugar portion (Table 2). The ^1^H NMR spectrum displayed the presence of the typical proton signals of two phenylpropanoid moieties. In particular, signals of two couple of trans-olefinic protons at δ 6.31 and 6.43 (each, d, *J* = 15.8 Hz) and at δ 7.69 and 7.64 (each, d, *J* = 15.8 Hz) were observed. Signals at δ 7.20 (d, *J* = 1.9 Hz), 7.09 (dd, *J* = 1.9, 8.3 Hz) and 6.82 (d, *J* = 8.3 Hz) confirmed the 1,3,4 substituted aromatic system of a feruloyl moiety along with a singlet signal at δ 6.89 (2H, s) ascribable to the 1,3,4,5 substituted aromatic system of a sinapoyl moiety (Figure 1, Figures S1–S8, Table 2).

The ^1^H NMR spectrum displayed a signal corresponding to an anomeric proton at δ 5.83 (d, *J* = 8.0 Hz) assigned to a glucose unit. A detailed analysis of HSQC, COSY and HMBC experiments allowed us to attribute the signal at δ 5.10 to H-2glc, downfield shifted for the involvement in an ester linkage. The HMBC experiment allowed us to assign the sequence and linkage sites of sugar moieties, due to the correlation peaks between the proton signal at δ 5.83 (H-1glc) and the carbon resonance at δ 166.8 (C-9′) and between the proton signal at δ 5.10 (H-2glc) and the carbon resonance at δ 167.1 (C-9″). On this basis, compound **16** was established as 1-*O*-feruloyl-2-*O*-sinapoyl-β-D-glucopyranoside, herein reported for the first time.

Compounds **5**–**7**, **11**–**15** and **17** were identified by analysis of spectroscopic data in comparison to those reported in the literature as methylsinapate (**5**) [6], 1-*O*-sinapoyl-β-D-glucopyranoside (**6**) [6], 1-*O*-feruloyl-β-D-glucopyranoside (**7**) [6], sinapic acid (**11**), 3,6′-*O*-disinapoylsucrose (**12**) [6], ferulic acid (**13**) [18], 1,2-*O*-disinapoyl-β-D-glucopyranoside (**15**) [6] and 3,4,6′-*O*-trisinapoylsucrose (**17**) [6]. 3-*O*-feruloyl-6′-*O*-sinapoylsucrose (**14**) was detected for the first time in this species (Figure 1).

### 3.3. Evaluation of the Antioxidant Activity of Different Extracts of Daikon Sprouts

To explore the possibility as to whether *R. sativus* sprouts are a suitable source of bioactives, the antioxidant activity of “green” extracts (EtOH, 70% EtOH/H_2_O and 50% EtOH/H_2_O) was evaluated and compared with that of the MeOH extract. Firstly, the phenolic content and radical scavenging activity of each extract were determined by colorimetric assays. The phenolic content was measured by the Folin–Ciocalteu method. The 70% EtOH/H_2_O extract showed the highest total phenolic content with a value of 400.95 mg GAE/g extract (Table S1).

The radical scavenging activity of the extracts was tested by the spectrophotometric DPPH and TEAC assays. The results showed the highest activity for 70% EtOH/H_2_O extract with a TEAC value of 1.95 mM, compared to the TEAC value of 1.81 mM exhibited by the positive control quercetin 3-*O*-β-D-glucopyranoside. All the other extracts showed radical scavenging activity with TEAC values ranging from 1.02 to 1.70 mM. The DPPH assay highlighted the highest scavenging activity for the 70% EtOH/H_2_O extract (IC50 93.97 µg/mL) (Table S1). In the literature, the antioxidant activity was reported for MeOH, CHCl_3_ and CH_3_COCH_3_ extracts of daikon sprouts [6,13]. Herein, the antioxidant activity exhibited by the “green” extracts represents the possibility to use these extracts as a nutraceutical ingredient.

### 3.4. Activation of the Nrf2 Pathway Assessed by a Reporter Gene Assay

**Figure 2:**
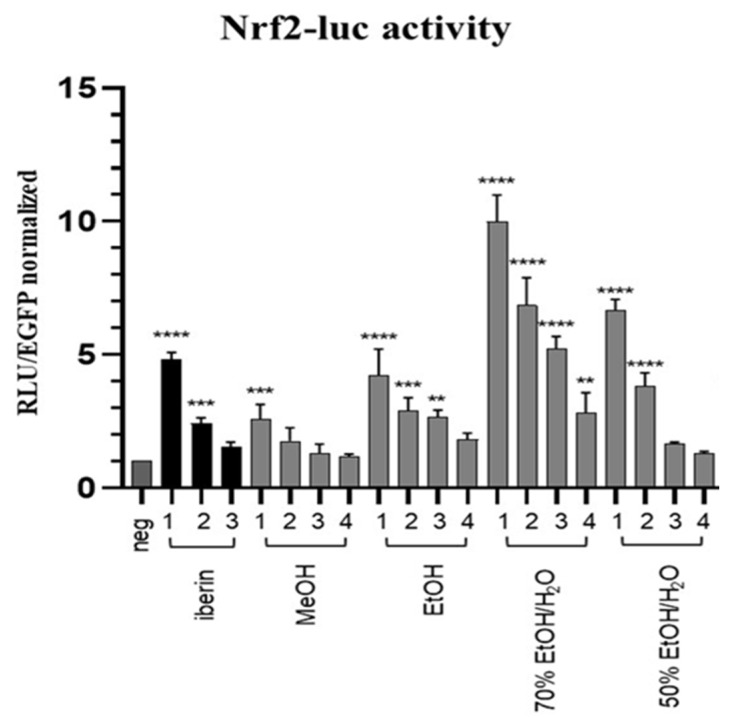
Activation of the Nrf2 pathway. HepG2-ARE Luc cells were treated with DMSO (0.1%, negative control,), Iberin (positive control); 3, 1.5, 0.75 µM (*x* values 1–3, respectively) and 30, 20, 10, 5 µg/mL (*x* values 1–4, respectively) of daikon sprouts extracts for 16 h. Luciferase expression was assessed, normalized to the cell count and expressed as fold induction of the negative DMSO control. The bar graph depicts compiled data of three independent experiments (means + SD). *p*-value ** <0.01; *** <0.001; **** <0.0001 (One-way ANOVA with Dunnett’s post hoc test vs. vehicle control).

The extracts of *R. sativus* sprouts were tested for induction of the Nrf2 pathway in a luciferase reporter gene assay. At concentrations of 30, 20, 10 and 5 µg/mL, the examined extracts did not elicit obvious cytotoxicity as the number of viable cells was in the range of 80–95% of the solvent control. Figure 2 shows the data for Nrf2 (ARE)-dependent luciferase induction. As in the DPPH and TEAC results for green extracts, the hydroalcoholic extracts (50% and 70% EtOH/H_2_O) showed pronounced bioactivtity and concentration-dependently induced Nrf2 activity. At 30 µg/mL they were more active (6.5- and 10-fold activation compared to control cells, respectively) than the iberin (3 µM; 5-fold activation) used as a positive control.

### 3.5. Multivariate Data Analysis

Multivariate data analysis has proved over time to be a powerful statistical technique, especially useful in processing large datasets when more than one observation is involved due to its effectiveness in making data plain. It is currently widely employed for several applications in industrial and scientific fields, such as control and optimization processes, quality control, research and development [21]. In the present study, the chemical metabolome of daikon sprouts was defined by employing different extraction protocols.

The extracts of daikon sprouts were submitted to ^1^H NMR experiments and then analyzed by an untargeted and targeted approach of multivariate data analysis. Specifically, the exploratory statistical data analysis by PCA and PLS-DA methods were applied to discriminate the samples. The raw data were first filtered by using MestreNova software and then processed by using SIMCA-P+ software.

#### 3.5.1. Untargeted Metabolite Profiling of *R. sativus* var *longipinnatus* Extracts

The ^1^H NMR spectra of extracts of *R. sativus* sprouts obtained with different solvents were compared (Figure S11). The careful analysis of ^1^H NMR spectra showed spectra with a lot of crowded signals; also, overlapping signals were displayed. In the ^1^H NMR spectra, it was possible to identify three regions: aliphatic, carbinol and aromatic proton regions (Figure S9). The first step was to perform the unsupervised PCA data analysis to obtain an overview on trends and outliers among the samples, and therefore, the NMR peak lists were obtained from the entire spectrum range of samples and by measuring the selected peak area in the ^1^H NMR spectra. The first and second components explained 72.1% and 21.4% of the variance, respectively. The PCA score plot allowed the separation of the analyzed samples into clusters, based on the different solvents used for fresh sprouts of *R. sativus*. A different distribution was observed for the extracts in the PCA score plot (Figure S11). In particular, the hydroalcoholic extracts of 50% and 70% EtOH/H_2_O (E50A1-3 and E50B1-3; E70A1-3 and E70B1-3) were separated from each other by the second principal component; the alcoholic extracts EtOH and MeOH (EA1-3 and EB1-3, MA1-3 and MB1-3) were defined in a second cluster, separated by the first principal component from the hydroalcoholic extracts. The untargeted PCA loading plot highlighted the signals responsible for the distribution on the PCA score plot. In particular, the loading plot showed that the EtOH extracts were characterized by the signals at δ 0.89, 0.93 and 1.33 ascribable to fatty acids (Figure S11B). The PCA loading plot (Figure S11B) showed many signals close to the origins, indicating their common presence in the extracts.

#### 3.5.2. Targeted Metabolite Profiling of *R. sativus* var *longipinnatus* Extracts

Moreover, a targeted PCA was performed with the aim of understanding how the different extraction protocols could influence the presence of the metabolites. ^1^H NMR metabolomics has the advantage of identifying the primary and specialized metabolites simultaneously. A detailed analysis of the proton spectra provided the signal characteristic of each metabolite, and the dataset for primary and specialized metabolites was reported in Table S2. Typical signals corresponding to aminoacids at δ 0.98 (d, *J* = 7.0 Hz) for valine, 1.00 (s) for isoleucine, 1.49 (d, *J* = 7.1 Hz) for alanine, 2.30 (t, *J* = 7.0 Hz) for GABA, 2.85 (dd, *J* = 17.3, 3.7) for aspartic acid and 6.86 (m) for tyrosine were evident [12,24]. In the ^1^H NMR spectra, proton signals at δ 2.50 (m) and 4.27 (dd, *J* = 8.0, 4.0) ascribable to the organic acids, succinic acid and malic acid, respectively, were observed. Lipids at δ 1.33 (m) and 5.38 (m) were correlated to fatty acids and polynsaturated fatty acids (PUFA), and also at δ 0.76, phytosterol was identified. The ^1^H NMR spectra displayed signals for anomeric protons related to β-glucose (δ 4.50, d, *J* = 8.0 Hz), α-glucose (δ 5.14, d, *J* = 3.6 Hz) and sucrose (δ 5.40, d, *J* = 3.8 Hz). Along with primary metabolites, glucosinolates such as sulfoxide glucosinolates (δ 2.70 brt, *J* = 7.1 Hz) and indolic glucosinoates (δ 7.24, s) were also identified. Proton signals for hydroxycinnamic acid derivatives were assigned. Typical signals at δ 5.82 (d, *J* = 8.3 Hz), 7.20 (d, *J* = 1.9 Hz), 7.50 (d, *J* = 15.8 Hz) and 7.54 (d, *J* = 15.8 Hz) were related to 1,2-*O*-disinapoyl-β-D-glucopyranoside (**15**), 1-*O*-feruloyl-2-*O*-sinapoyl-β-D-glucopyranoside (**16**), 3,4,6′-*O*-trisinapoylsucrose (**17**) and sinapic acid (**11**), respectively. The proton signal at δ 7.71 (d, *J* = 15.8 Hz) was assigned to 3,6’-*O*-disinapoylsucrose (**12**) and 3-*O*-feruloyl-6′-*O*-sinapoylsucrose (**14**) while the signal at δ 7.74 (d, *J* = 15.8 Hz) was assigned to 1-*O*-sinapoyl-β-D-glucopyranoside (**6**) and 1-*O*-feruloyl-β-D-glucopyranoside (**7**). The analysis of the 2D NMR spectra of the extracts and the comparison of their ^1^H NMR spectra with those of the isolated compounds afforded the clear identification of the metabolites in the proton NMR spectra.

So, PCA was performed by measuring the selected peak area for each identified metabolite in the ^1^H NMR dataset (Figure S10, Table S2). The PCA was used to acquire a general insight and visualize any relation (trends and outliers) among the observations (samples).

**Figure 3:**
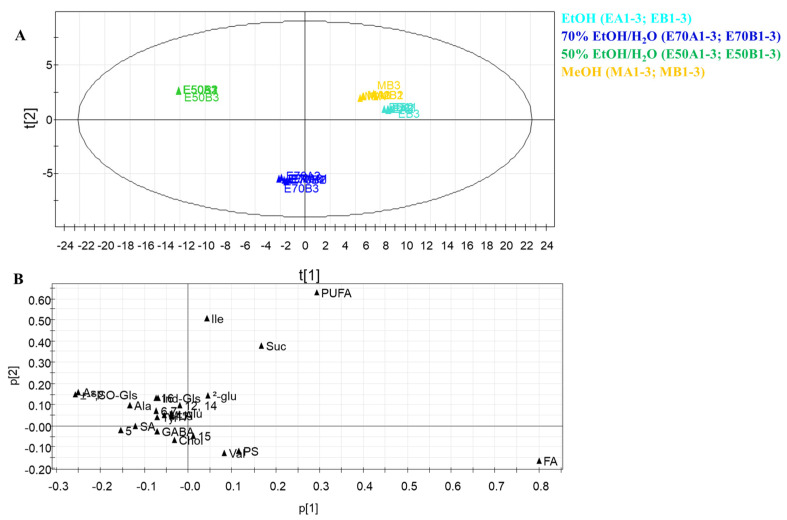
(**A**) Principal component analysis of *R. sativus* extracts. PCA score scatter plot, (**B**) PCA loading plot. Two batches (**A**,**B**) in triplicate, (1–3) for each extract. (Ala) Alanine; (Asp) Aspartic acid; (α-Glu) α-glucose; (β-Glu) β-glucose; (Chol) choline; (FA) Fatty acids; (GABA) gamma-aminobutyric acid; (PS) phytosterols; (Ile) Isoleucine; (Ind-Gls) indolic glucosinolates; (MLA) Malic acid; (PUFA) polyunsaturated fatty acids; (SA) Succinic acid; (SO-Gls) sulfoxide glucosinolates; (Suc) sucrose; (Tyr) tyrosine, (Val) Valine; (**5**) methylsinapate; (**6**, **7**) 1-*O*-Sinapoyl-β-D-glucopyranoside and 1-*O*-Feruloyl-β-D-glucopyranoside; (**11**) sinapic acid; (**12**, **14**) 3-*O*-feruloyl-6′-*O*-sinapoyl-sucrose and 3-*O*-sinapoyl-6′-*O*-sinapoyl-sucrose; (**15**) 1,2-*O*-disinapoyl-β-D-glucopyranoside; (**16**) 1-*O*-Feruloyl-2-*O*-sinapoyl-β-D-glucopyranoside; (**17**) 3,4-*O*-disinapoyl-6′-*O*-sinapoyl-sucrose.

The resulting model showed good fitness and the absence of outliers. PC1 and PC2 contributed to 84.0% and 13.3% of the variance, respectively. Therefore, the extracts were well discriminated against each other. In the targeted PCA model, a clear difference between extracts was evident, probably due to solvents of different polarity affecting the metabolite content. The extracts obtained with MeOH and EtOH appeared located very close in a section of the score plot, far from the others. Specifically, in the PCA, the MeOH and EtOH (each, two batches in triplicate) were separated from EtOH/H_2_O extracts along with the first principal component (PC1), showing how the presence of water could affect the extraction process (Figure 3). The 70% EtOH/H_2_O extracts were separated from the 50% EtOH/H_2_O extracts along with the second principal component (PC2). The targeted PCA loading plot highlighted how MeOH and EtOH extracts showed higher concentrations of primary metabolites such as PUFA and sucrose. With regard to “green” extracts, the amount of glucosinolates was higher in 50% EtOH/H_2_O than in 70% EtOH/H_2_O extract, while this latter was characterized by a higher occurrence of GABA and choline (Figure 3).

#### 3.5.3. Partial Least Square Discriminant Analysis (PLS-DA)

PLS-DA was performed to observe the relationship between the chemical profiles of the extracts and their bioactivity profiles. The components one and two in the PLS-DA score plot explained 83.4% and 13.3% of the variation, respectively, exhibiting a good separation between these groups. The PLS-DA analysis showed a distinct separation (R2Y, 0.97) and good predictability (Q2, 0.61).

**Figure 4:**
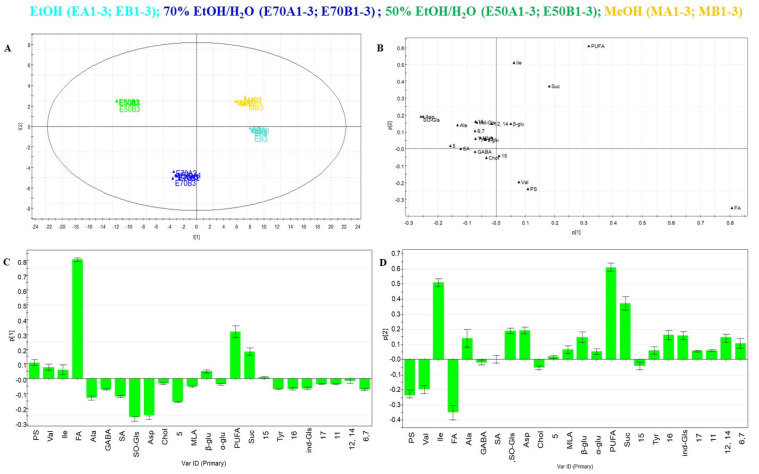
(**A**) PLS-DA correlation of ^1^H NMR data matrix with Nrf2 activity (measured by a gene reporter assay at 30 µg/mL). (**B**) PLS-DA Score Plot, (**C**) single variables to the principal component 1 (Comp. 1), Y = primary and specialized metabolites. (**D**) Single variables to the principal component 2 (Comp. 2), Y = primary and specialized metabolites. Two batches (**A**,**B**) in triplicate, (1–3) for each extract. (Ala) Alanine; (Asp) Aspartic acid; (α-Glu) α-glucose; (β-Glu) β-glucose; (Chol) choline; (FA) Fatty acids; (GABA) gamma-aminobutyric acid; (PS) phytosterols; (Ile) Isoleucine; (Ind-Gls) indolic glucosinolates; (MLA) Malic acid; (PUFA) polyunsaturated fatty acids; (SA) Succinic acid; (SO-Gls) sulfoxide glucosinolates; (Suc) sucrose; (Tyr) tyrosine, (Val) Valine; (**5**) methylsinapate; (**6**,**7**) 1-*O*-Sinapoyl-β-D-glucopyranoside and 1-*O*-Feruloyl-β-D-glucopyranoside; (**11**) sinapic acid; (**12**, **14**) 3-*O*-feruloyl-6′-*O*-sinapoyl-sucrose and 3-*O*-sinapoyl-6′-*O*-sinapoyl-sucrose; (**15**) 1,2-*O*-disinapoyl-β-D-glucopyranoside; (**16**) 1-*O*-Feruloyl-2-*O*-sinapoyl-β-D-glucopyranoside; (**17**) 3,4-*O*-disinapoyl-6′-*O*-sinapoyl-sucrose.

PLS-DA showed a good separation among the extracts along with the first principal component (PC1). The hydroalcoholic extracts were separated from MeOH and EtOH extracts by PC1 but not by the PC2 component. The loading scatter plot showed potentially distinct metabolites based on contributions and reliability to the separation observed in the score scatter plot (Figure 4B). As previously described, metabolites in the loading plot that were distant from the origin could be considered markers of the extraction procedures as a confirmation of their different distribution in different samples. The specific contribution of single variables to PC1 is reported in Figure 4C. Considering the PLS-DA column loading plot of first variable, the metabolites marked below the baseline were present at higher concentrations in the extracts obtained by hydroalcoholic maceration. The hydroalcoholic maceration protocol yielded higher concentrations of primary metabolites such as the amino acids alanine (Ala), aspartic acid (Asp), and tyrosine (Tyr) and some of the specialized metabolites such as sulfoxide glucosinolates (SO-Gls), indolic glucosinolates (Ind-Dls) and hydroxycinnamic acid derivatives **5**–**7**, **11**–**12**, **14** and **17** along with the afore-described compound **16**. Thus, it was possible to deduce that metabolites such as glucosinolates (SO-Gls and Ind-Gls) and hydroxycinnamic acid derivatives contributed to a higher Nrf2 induction activity of the hydroalcoholic extracts.

Along with the occurrence of glucosinolates reported to activate the Nrf2 pathway [31], PLS-DA analysis highlighted the contribution of the hydroxycinnamic acid derivatives **6**, **7**, **11**, **12**, **14**, **16** and **17** to the higher activity of the hydroalcoholic extracts in terms of Nrf2 activation. In particular, compounds 1-*O*-feruloyl-2-*O*-sinapoyl-β-D-glucopyranoside (**16**) and 3-*O*-feruloyl-6′-*O*-sinapoylsucrose (**14**) were good discriminants for the increased activity as shown in the PLS-DA Score Plot (Figure 4).

All the statistical data highlighted the ability of EtOH/H_2_O mixtures to extract specialized metabolites responsible for the bioactivity of *Raphanus sativus.* This behaviour can be ascribable to the water, for its ability to swell the plant material, and to ethanol, responsible for breaking the binding between the solutes and plant matrix, facilitating mass transfer of the metabolites [32,33]. The mixtures of EtOH/H_2_O, modulating the polarity of the solvent, showed a better synergistic effect for the extraction of bioactive compounds.

## 4. Conclusions

The sprouts of *Raphanus sativus* var *longipinnatus* represent a functional food due to their high nutritional value and health-promoting effects. In this context, daikon sprouts show interesting antioxidant properties. The obtained results provide a comprehensive chemical investigation of these microgreens highlighting how the extraction process can influence the presence of bioactive metabolites and the antioxidant properties of daikon-based extracts. Among all the extracts, the 50% EtOH/H_2_O is characterized by a higher content of glucosinolates as well as of 1-*O*-sinapoyl-β-D-glucopyranoside (**6**) and 1-*O*-feruloyl-β-D-glucopyranoside (**7**). This is in agreement with literature data reporting a synergistic effect in the extraction of bioactive compounds achieved by adding water to ethanol, increasing the permeability of plant tissues and enabling better mass transfer by diffusion [33].

The obtained results provide a comprehensive chemical investigation of these microgreens and, at the same time, highlight how the extraction process can influence the presence of bioactive metabolites. Moreover, the obtained data show how the extraction process can also affect the antioxidant properties of daikon-based extracts. The investigation of daikon sprouts afforded the identification of p-hydroxycinnamic acid derivatives, glucosinolates and oxylipins; in particular, 1-*O*-feruloyl-2-*O*-sinapoyl-β-D-glucopyranoside (**16**), a new p-hydroxycinnamic acid derivative, was here unambiguously characterized by NMR analysis for the first time. Moreover, benzoylglucoraphenin (**10**), a glucosinolate never reported previously, was putatively identified by LC-MS analysis. Here, the combination of the LC-MS and NMR analysis provided a complete fingerprint of daikon sprouts, taking into account both primary and specialized metabolites. Diet provides a wide variety of bioactive nutrients that possess beneficial effects to health and are able to activate the Nrf2 signalling pathway [14], further confirmed by biological evaluation here assayed. The antioxidant activity shown by the extracts of daikon sprouts encourages the use of daikon sprouts as a functional food. “Green” extracts exerted antioxidant activity by both scavenging radicals and activating Nrf2 signalling in cells.

The analysis of the PLS-DA model shows correlations between the chemical constituents observed in NMR fingerprints and biological activities (Nrf2 activation). In particular, the contribution of hydroxycinnamic acid derivatives (**6**, **7**, **11**, **12**, **14**, **16** and **17**) to the higher activity of the hydroalcoholic extracts in stimulating the activation of Nrf2 became evident.

In conclusion, the results of the antioxidant activity make *R. sativus* sprouts, processed by different extraction methods, a rich source of phytochemicals with potential health benefits. In conclusion, the antioxidant activity exhibited by *R. sativus* sprouts, processed by different extraction solvents, confirms this food as a rich source of phytochemicals with potential health benefits.

## Figures and Tables

**Table 2:** ^13^C and ^1^H NMR data of compound **16** (600 MHz, *δ* ppm, in CD_3_OD).

16		
	δ_C_	δ_H_ (*J* in Hz)
glucose		
1	94.0	5.83 (d, 8.0)
2	74.3	5.10 (dd, 8.0, 9.0)
3	76.8	3.74 (dd, 9.0, 9.0)
4	71.2	3.54 (dd, 9.0; 9.0)
5	79.2	3.55 (m)
6	62.2	3.93 (dd, 12.0, 2.5)
		3.77 (dd, 12.0, 4.5)
1-*O*-feruloyl moiety		
1′	127.9	
2′	111.7	7.20 (d, 1.9)
3′	150.3	
4′	147.6	
5′	116.8	6.82 (d, 8.3)
6′	124.7	7.09 (dd,1.9; 8.3)
7′	149.4	7.69 (d, 15.8)
8′	116.3	6.31 (d, 15.8)
9′	166.8	
OCH_3_	56.8	3.90 s
2-*O*-sinapoyl moiety		
1″	126.2	
2″	106.9	6.89 s
3″	148.9	
4″	139.1	
5″	148.9	
6″	106.9	6.89 s
7″	148.5	7.64 (d, 15.8)
8″	115.5	6.43 (d, 15.8)
9″	167.1	
OCH_3_	56.7	3.87 s

## Data Availability

The data presented in this study are available within the article and its Supplementary Material.

## Supplementary Materials

The following supporting information can be downloaded at: https://www.mdpi.com/article/10.3390/antiox12081542/s1, Figure S1: HRMS/MS spectra of compound **10**; Figures S2–S8: NMR spectra of compound **16**; Figures S9 and S10: ^1^H NMR spectra of extracts of *R. sativus* sprouts; Figure S11: Principal component analysis of *R. sativus* extracts obtained by untargeted analysis; Table S1: Total Phenolic Content, DPPH^•^ and ABTS^•+^ radical scavenging activity of polar extracts of daikon sprouts; Table S2: Characteristic 1H NMR peaks identified in *R. sativus.*
