# Supplementary material for: LC-MS- and 1H NMR-Based Metabolomics to Highlight the Impact of Extraction Solvents on Chemical Profile and Antioxidant Activity of Daikon Sprouts (Raphanus sativus L.)
Source: Antioxidants (Basel). 2023 Aug 1;12(8):1542. doi: 10.3390/antiox12081542 (PMC10451950; doi:10.3390/antiox12081542)
Supplement: Supplementary file 1 [file antioxidants-12-01542-s001.zip › antioxidants-2515589-supplementary.pdf]

Supplementary Material

LC-MS- and ^{1}H NMR-based metabolomics to highlight the impact of extraction solvents on chemical profile and antioxidant activity of daikon sprouts (*Raphanus sativus* L.)

Ciro Cannavacciuolo [1,2,a], Antonietta Cerulli [1,a], Verena M. Dirsch [3], Elke H. Heiss [3], Milena Masullo [1], Sonia Piacente [1,*]

[a]*Dipartimento di Farmacia, Università degli Studi di Salerno, via Giovanni Paolo II n. 132, 84084 Fisciano (SA), Italy*

[b]*PhD Program in Drug Discovery and Development, Università degli Studi di Salerno, via Giovanni Paolo II n. 132, I-84084 Fisciano, SA, Italy.*

[c]*Department of Pharmaceutical Sciences, University of Vienna, Althanstrasse 14, 1090 Vienna, Austria*

[1] *contributed equally.*

[*] Corresponding author: Tel.: +39 089969763; Fax:+39 089969602

E-mail addresses: piacente@unisa.it

List of Supporting Information

Figure S1. HRMS/MS spectra of compound **10**.

Figure S2. ^{1}H NMR Spectrum (600 MHz, CD_3OD) of compound **16**.

Figure S3. ^{13}C Spectrum (150 MHz, CD_3OD) of compound **16**.

Figure S4. 1D-TOCSYspectrum (600 MHz, CD_3OD) of compound **16**.

Figure S5. HSQC Spectrum (CD_3OD) of compound **16**.

Figure S6 HMBC Spectrum (CD_3OD) of compound **16**.

Figure S7. COSY Spectrum (CD_3OD) of compound **16**.

Figure S8. ROESY Spectrum (CD_3OD) of compound **16**.

Table S1. Total Phenolic Content, DPPH$^{\bullet}$ and ABTS$^{\bullet+}$ radical scavenging activity of polar extracts of daikon sprouts

Table S2. Characteristic ^{1}H NMR peaks identified in *R. sativus*.

Figure S9. ^{1}H NMR spectra of different extracts of *R.sativus* sprouts

Figure S10. ^{1}H NMR spectrum of MeOH extract of *R. sativus* sprouts with primary and specialized metabolites.

Figure S11. Principal component analysis of *R. sativus* extracts obtained by untargeted analysis. (**A**) PCA score catter plot; (**B**) PCA loading plot.

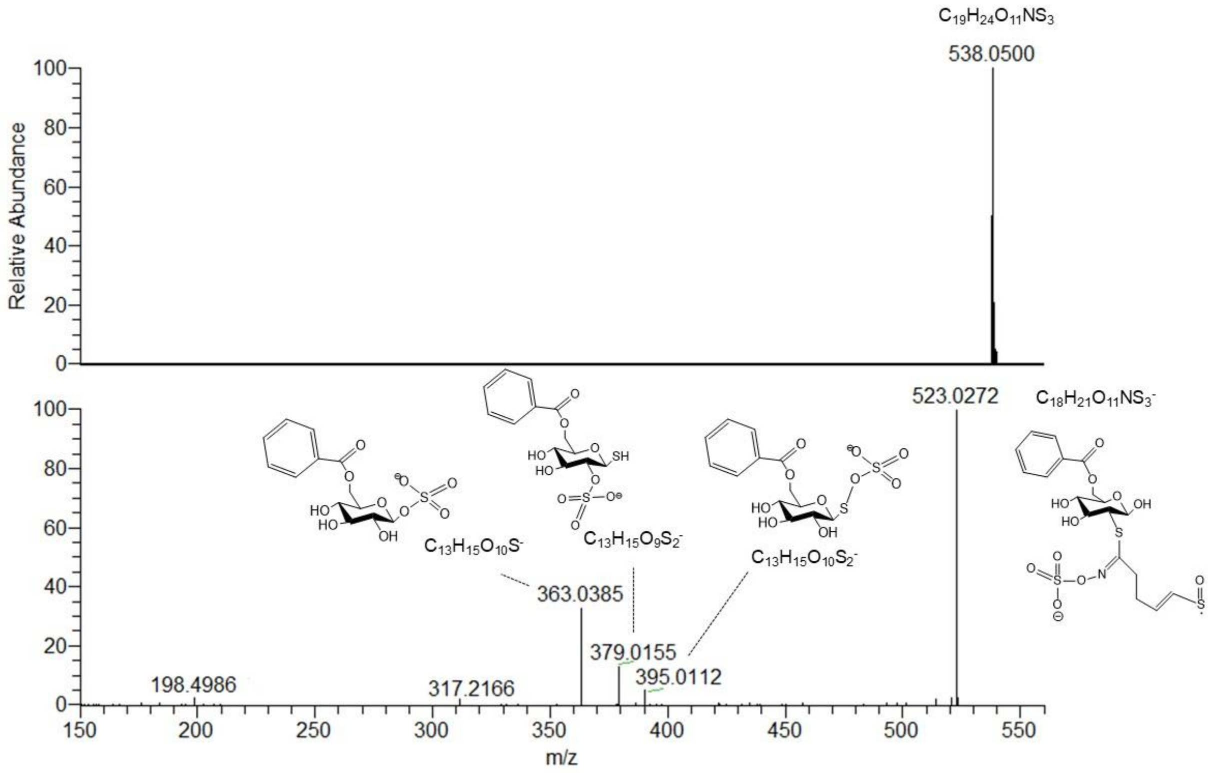

Fig. S1. HRMS/MS spectra of compound **10**

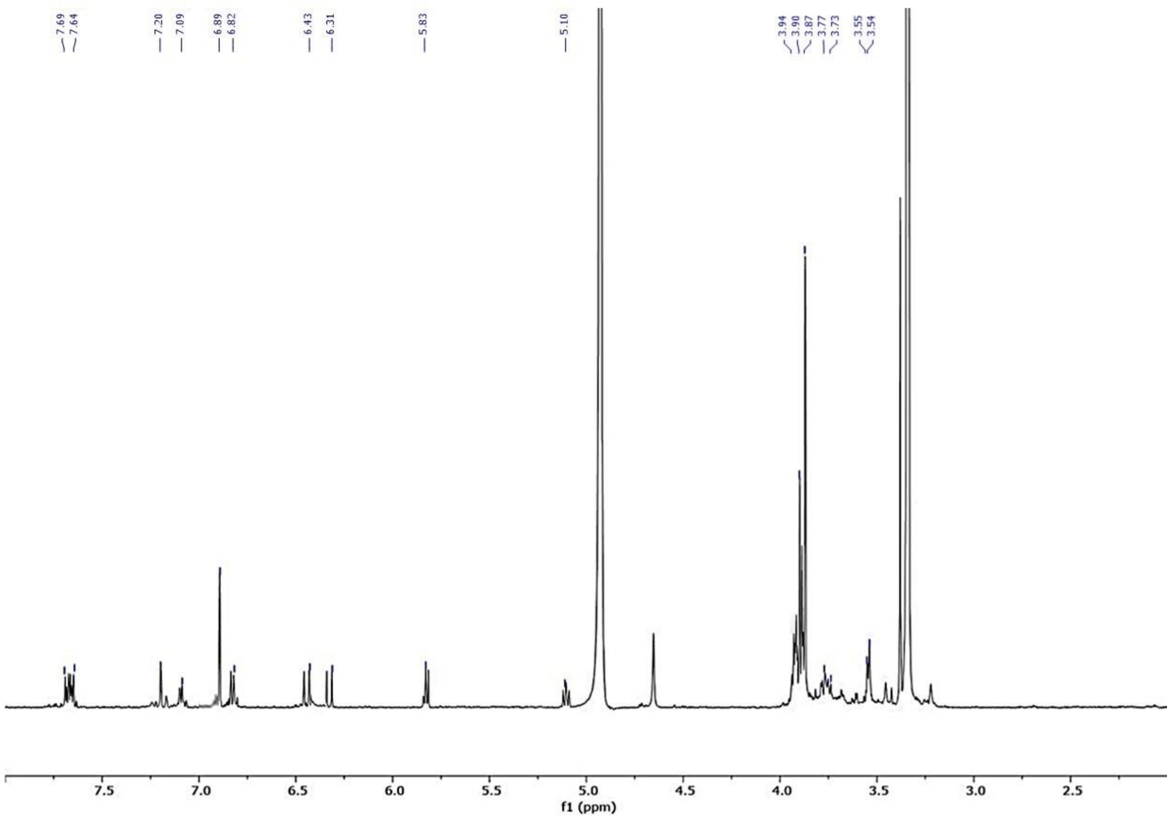

Fig. S2. ^{1}H NMR Spectrum (600 MHz, CD_3OD) of compound **16**.

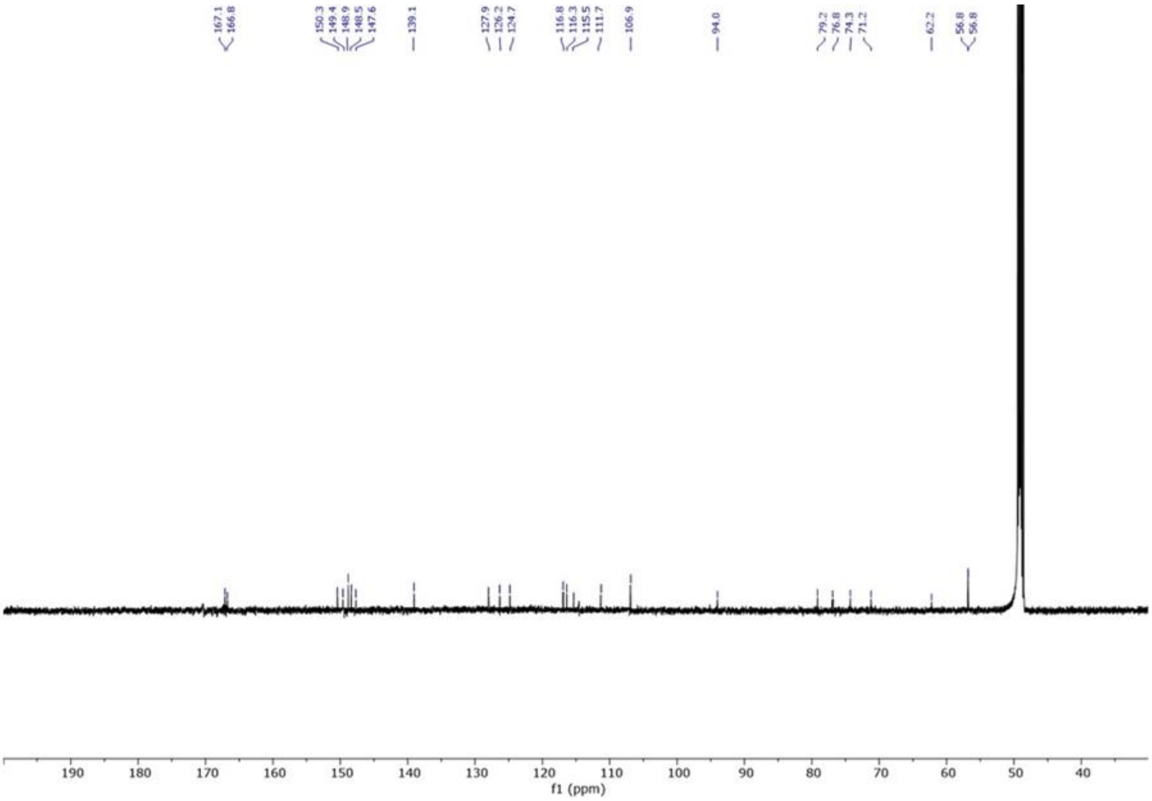

Fig. S3. ^{13}C Spectrum (150 MHz, CD_3OD) of compound **16**.

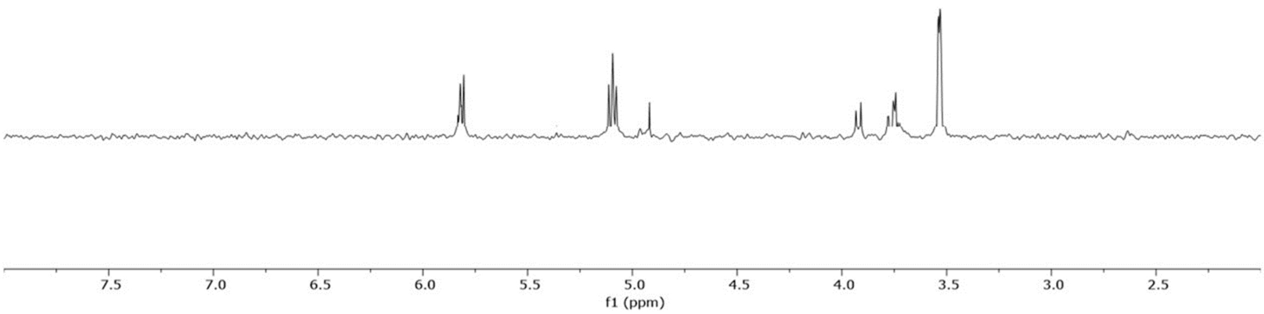

Fig. S4. 1D-TOCSYspectrum (600 MHz, CD_3OD) of compound **16**.

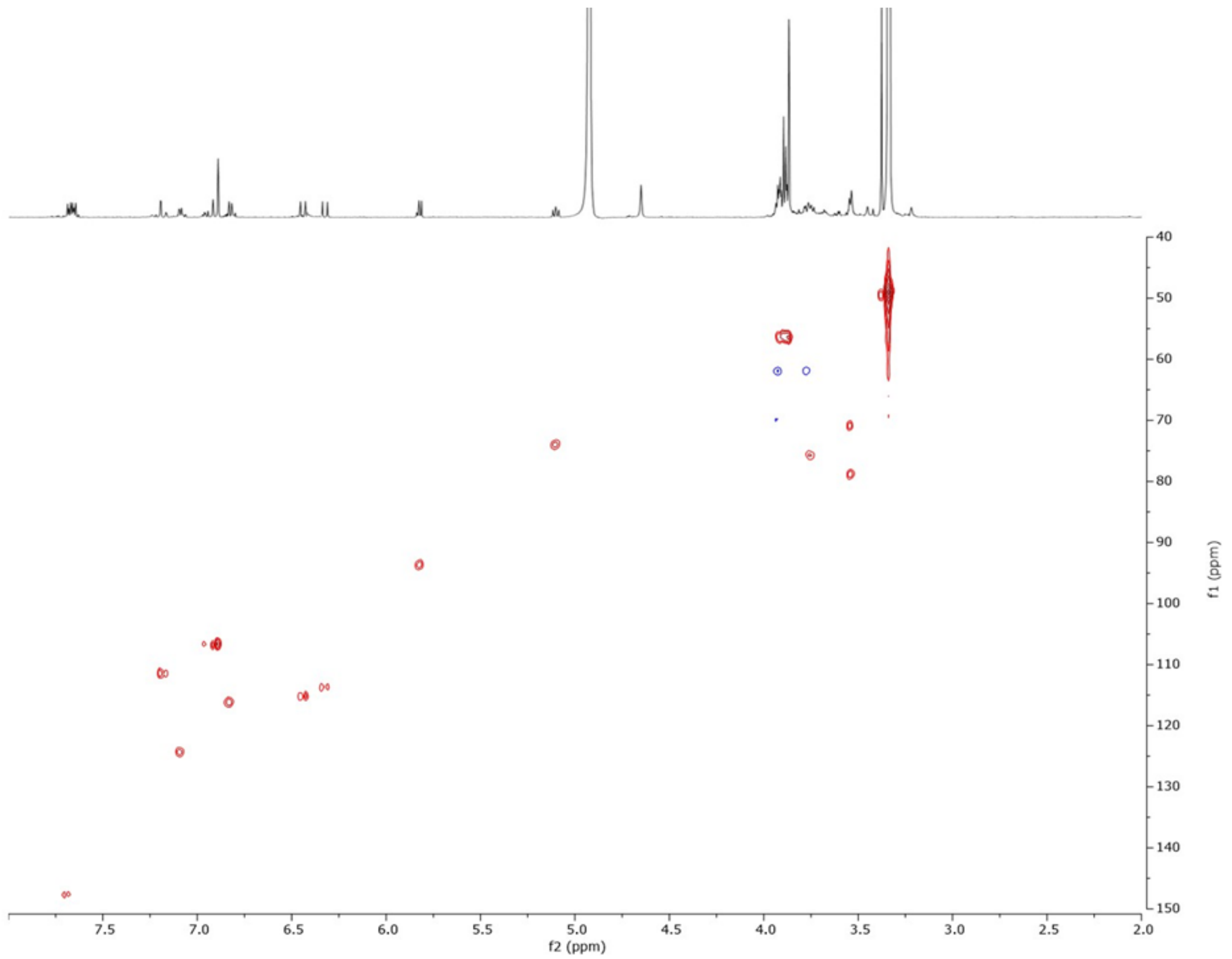

Fig. S5. HSQC Spectrum (CD_3OD) of compound **16**.

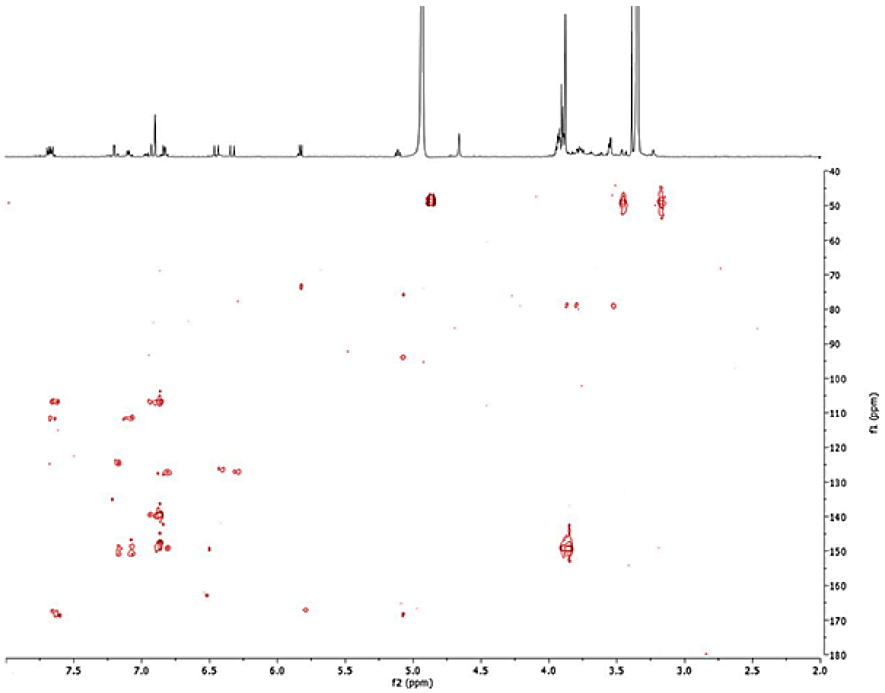

Fig. S6. HMBC Spectrum (CD_3OD) of compound **16**.

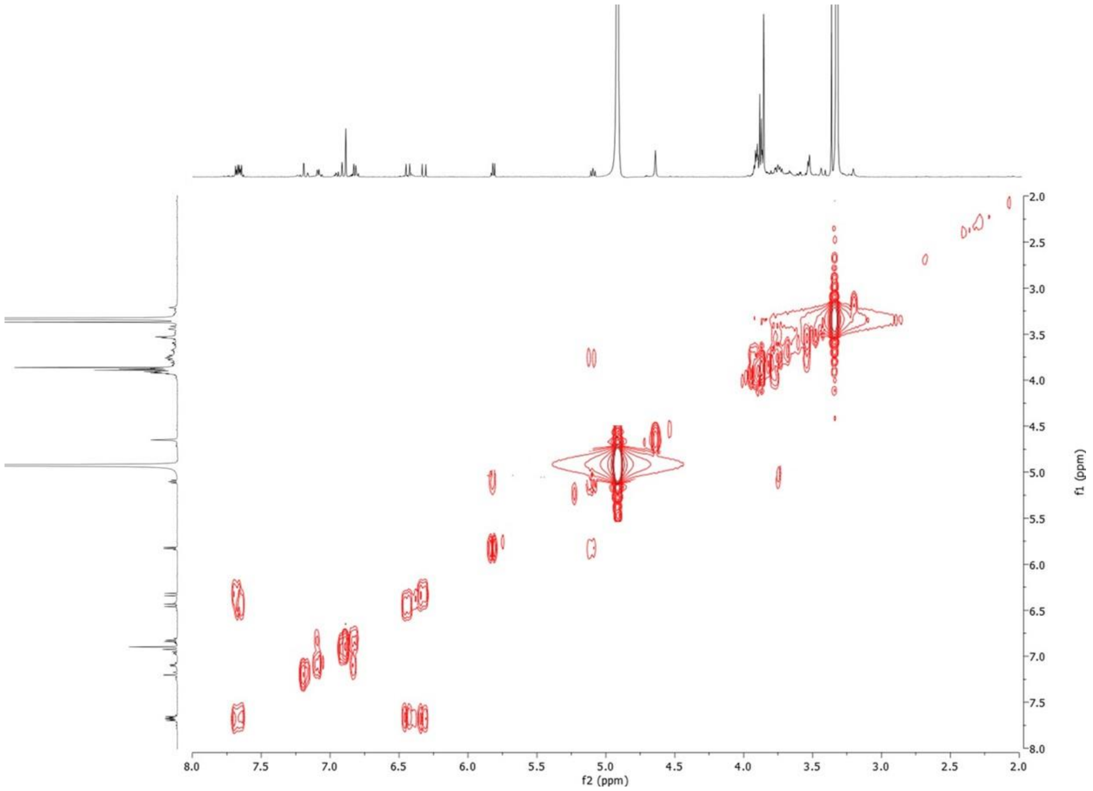

Fig. S7. COSY Spectrum (CD_3OD) of compound **16**.

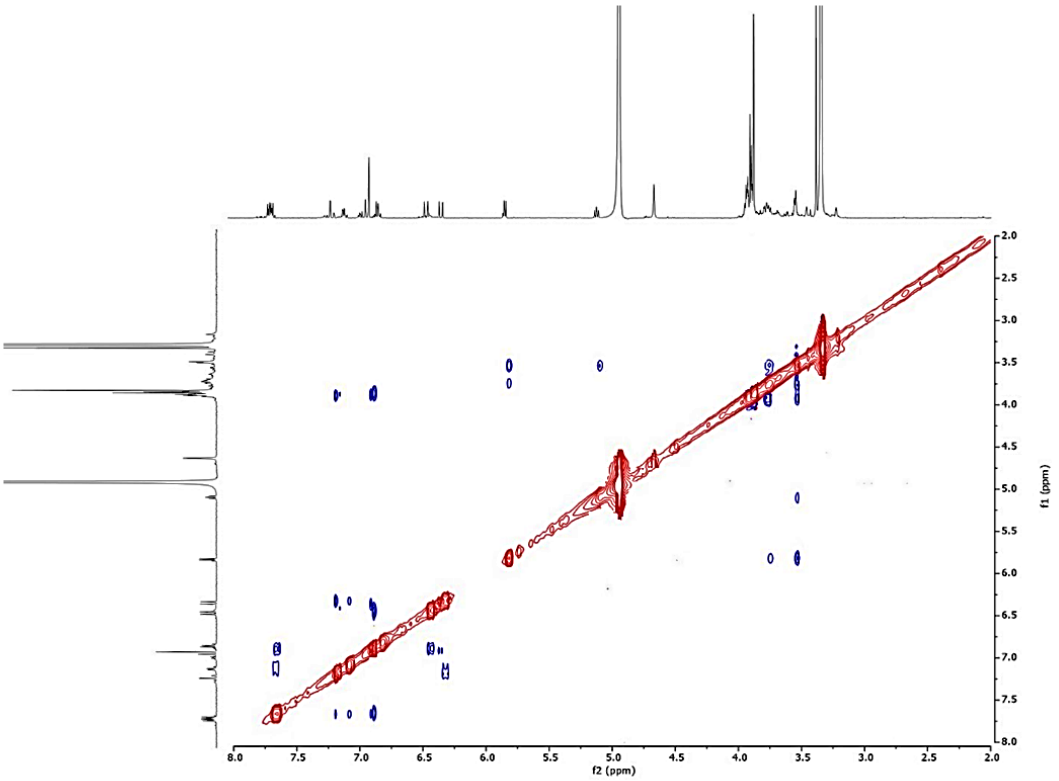

Fig. S8. ROESY Spectrum (CD_3OD) of compound **16**.

Table S1. Total Phenolic Content, DPPH•·and ABTS•+ radical scavenging activity of polar extracts of daikon sprouts

EXTRACTS	Total Phenolic Content [a] (GAE[A] ± SD)	DPPH• [b] (IC_{50}, μg/mL ± SD)	ABTS•+ [c] (TEAC[B] ± SD)
MeOH	269.36 ± 0.11	216.32 ± 0.12	1.02 ± 0.21
EtOH	283.56 ± 0.13	122.43 ± 0.12	1.70 ± 0.13
70% EtOH/H_2O	400.95 ± 0.33	93.97 ± 0.19	1.95 ± 0.14
50% EtOH/H_2O	322.40 ± 0.28	119.93 ± 0.18	1.34 ± 0.12
Ascorbic acid	-	4.53±0.01μM	-
Quercetin 3-O-gluc	-	-	1.81 ± 0.19 mM

[a]Values are expressed as gallic acid equivalent (GAE) mg/g of dried extract. [b]Values are expressed as micrograms per milliliter (μg/mL); [c]Values are expressed as concentration (mM) of a standard Trolox solution exerting the same antioxidant activity of a 1 mg/mL solution of texted extract.

Table S2. Characteristic ^{1}H NMR peaks identified in *R. sativus*.

Compound	^{1}H chemical shifts (multiplicity, in Hz)
Phytosterol (PS)	0.78 (m)
Valine (Val)	0.98 (d, 7.0)
Isoleucine (Ile)	1.01 (s)
Fatty Acids (FA)	1.33 (m)
Alanine (Ala)	1.49 (d, 7.1)
gamma-Aminobutyric acid (GABA)	2.30 (t, 7.3)
Succinic acid (SA)	2.50 (m)
Sulfoxide glucosinolates (SO-Gls)	2.70 (bt, 7.1)
Aspartic acid (Asp)	2.85 (dd, 17.3, 3.7)
Methylsinapate (**5**)	3.82 (s)
Malic acid (MLA)	4.27 (dd, 4.0, 8.0)
β-glucose (β-glc)	4.50 (d, 8.0)
α-glucose (α-glc)	5.14 (d, 3.6)
Polyunsaturated fatty acids (PUFA)	5.38 (m)
Sucrose (Suc)	5.40 (d, 3.8)
1,2-*O*-Disinapoyl-β-D-glucopyranoside (**15**)	5.82 (d, 8.3)
Tyrosine (Tyr)	6.86 (m)
1-*O*-Feruloyl-2-*O*-sinapoyl-β-D-glucopyranoside (**16**)	7.20 (d, 1.9)
Indolic glucosinoates	7.24 (s)
3,4,6'-*O*-Trisinapoylsucrose (**17**)	7.50 (d, 15.8)
Sinapic acid (**11**)	7.54 (d, 15.8)
3-*O*-Sinapoyl-6'-*O*-sinapoyl-sucrose (**12**), 3-*O*-Feruloyl-6'-*O*-sinapoyl-sucrose (**14**)	7.71 (d, 15.8)
1-*O*-Sinapoyl-β-D-glucopyranoside (**6**), 1-*O*-Feruloyl-β-D-glucopyranoside (**7**)	7.74 (d, 15.8)

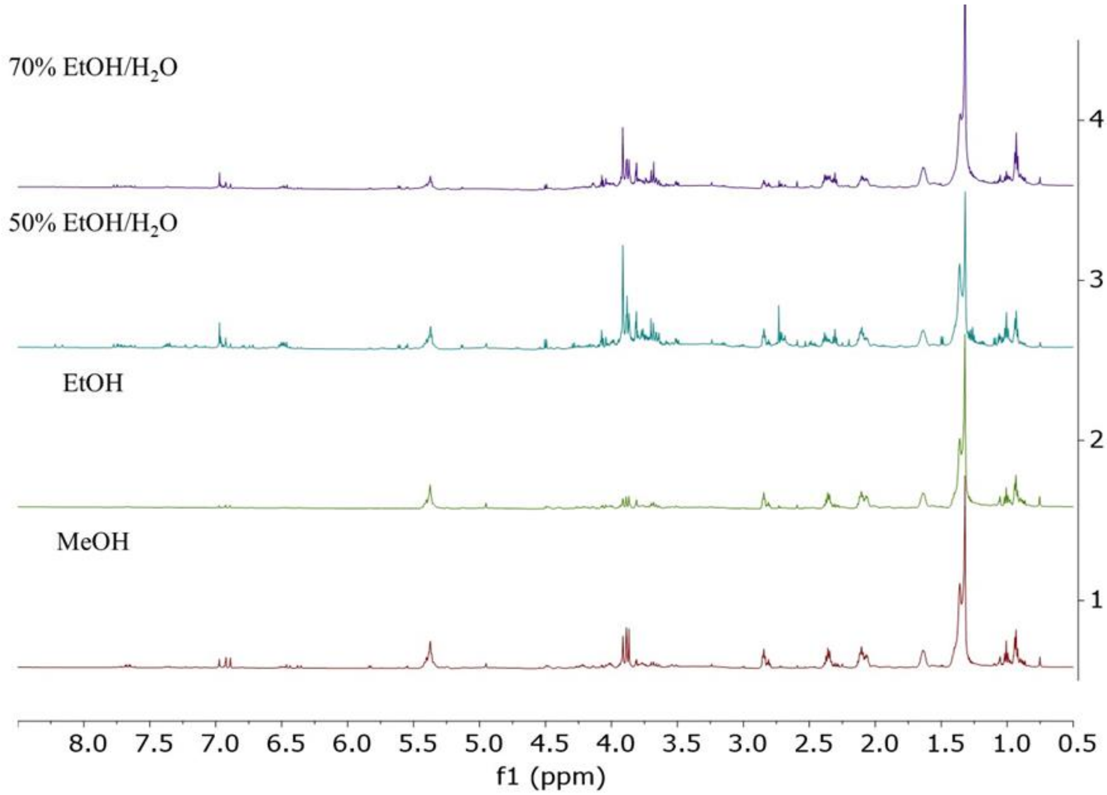

Fig. S9. ^{1}H NMR spectra of different extracts of *R.sativus* sprouts

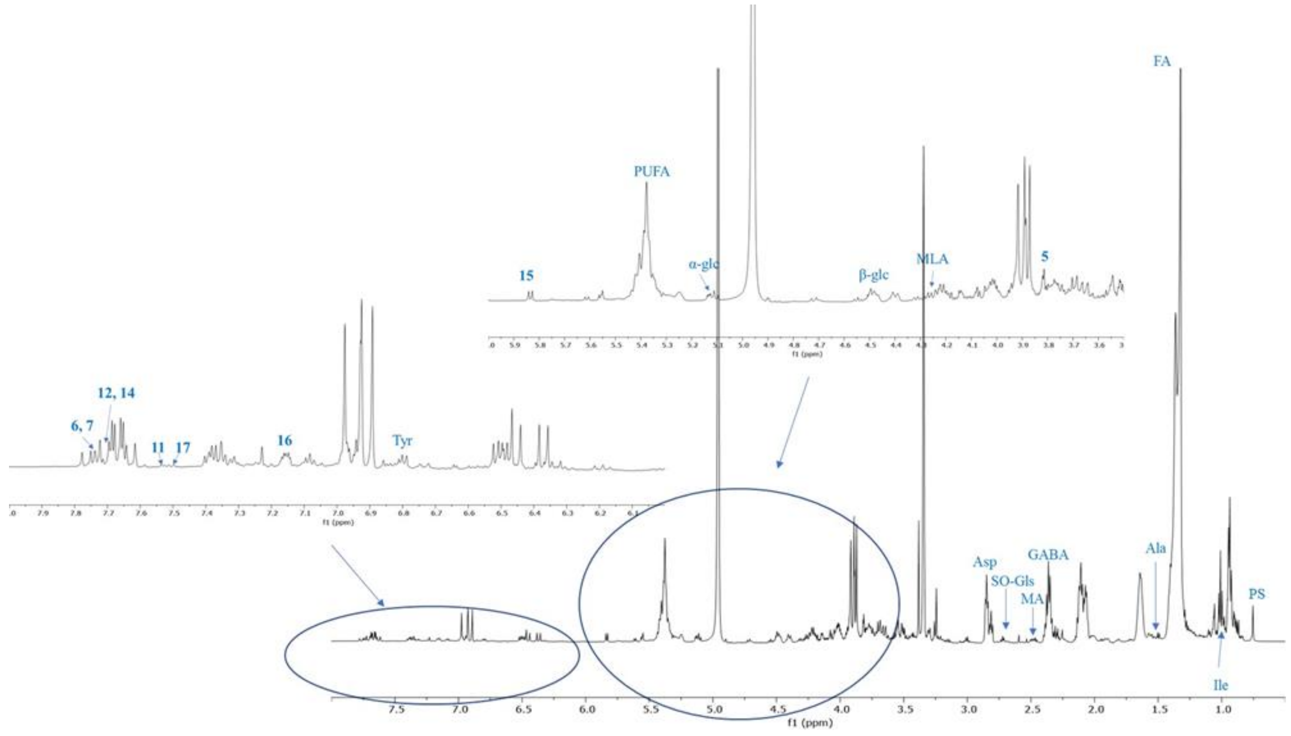

Fig. S10. ^{1}H NMR spectrum of MeOH extract of *R.sativus* sprouts with primary and specialized metabolites.

Legend: (Ala) Alanine; (Asp) Aspartic acid; (α-Glu) α-glucose; (β-Glu) β-glucose; (Chol) choline; (FA) Fatty acids; (GABA) gamma-aminobutyric acid; (PS) phytosterols; (Ile) Isoleucine; (Ind-Gls) indolic glucosinolates; (MLA) Malic acid; (PUFA) polyunsaturated fatty acids; (SA) Succinic acid; (SO-Gls) sulfoxide glucosinolates; (Suc) sucrose; (Tyr) tyrosine, (Val) Valine; (**5**) methylsinapate; (**6**,**7**) 1-*O*-Sinapoyl-β-D-glucopyranoside and 1-

O-Feruloyl-β-D-glucopyranoside; (**11**) sinapic acid; (**12**, **14**) 3-*O*-feruloyl-6'-O-sinapoyl-sucrose and 3-*O*-sinapoyl-6'-*O*-sinapoyl-sucrose; (**15**) 1,2-*O*-disinapoyl-β-D-glucopyranoside; (**16**) 1-O-Feruloyl-2-O-sinapoyl-β-D-glucopyranoside; (**17**) 3,4-*O*-disinapoyl-6'-*O*-sinapoyl-sucrose.

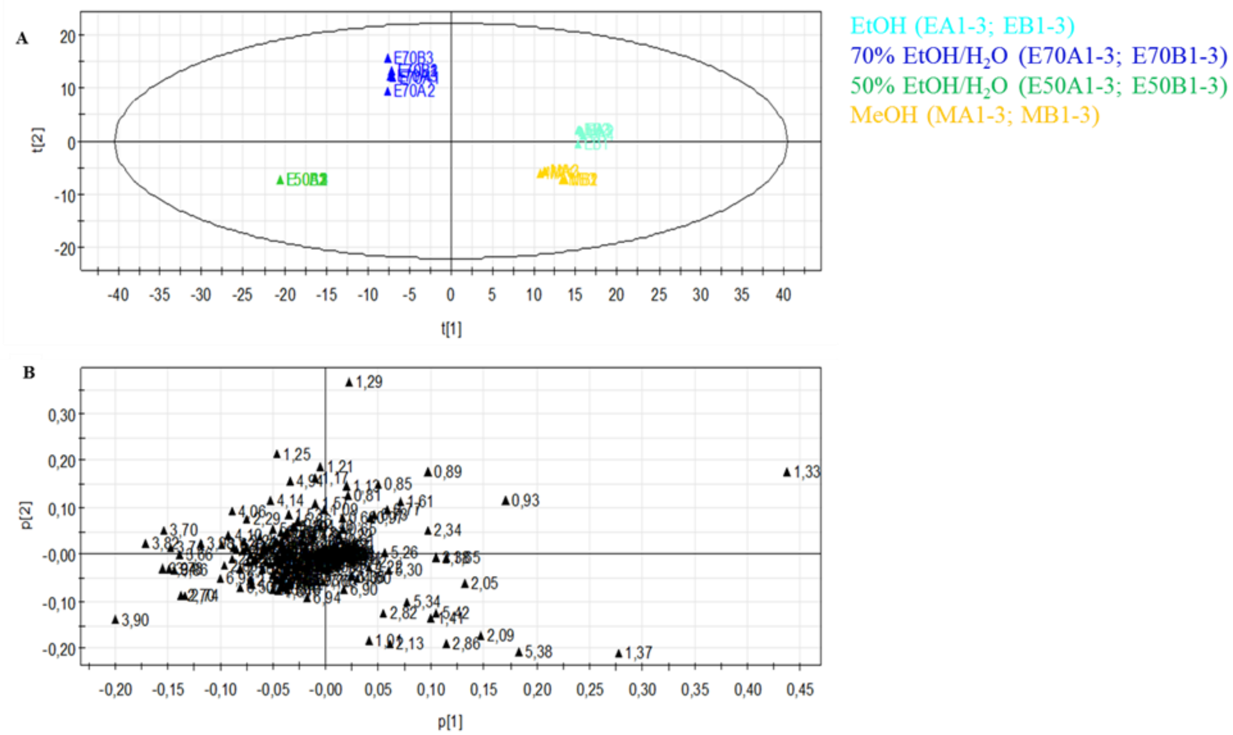

Fig. S11. Principal component analysis of *R. sativus* extracts obtained by untargeted analysis. (**A**) PCA score catter plot; (**B**) PCA loading plot.
